# High-resolution metagenomic characterization of gut microbiota composition and functional pathways in irritable bowel syndrome

**DOI:** 10.1038/s41598-026-52163-w

**Published:** 2026-05-20

**Authors:** Purnika Damindi Ranasinghe, Nawroz Barazanji, Olga Bednarska, Malin Bergman Jungeström, Peter Lundberg, Åsa V. Keita, Susanna Walter, Rozalyn Simon

**Affiliations:** 1 Pathogen Genomics, Bacteriology Branch, Veterinary Sciences Division, Agrifood and Biosciences Institute, Belfast, UK; 2 Clinical Department of Gastroenterology and Hepatology, Region Östergötland, Linköping, Sweden; 3 Clinical Department of Precision Medicine Laboratory, Region Östergötland, Linköping, Sweden; 4 Department of Health, Medicine and Caring Sciences, Linköping University, Linköping, Sweden; 5 Clinical Department of Medical Radiation Physics, Region Östergötland, Linköping, Sweden; 6 Clinical Department of Radiology in Linköping, Region Östergötland, Linköping, Sweden; 7 Center for Medical Image Science and Visualization (CMIV), Linköping University, Linköping, Sweden; 8 Department of Biomedical and Clinical Sciences, Linköping University, Linköping, Sweden; 9 Present Address: Department of Medicine, Karolinska Institute, Stockholm, Sweden

**Keywords:** Irritable bowel syndrome, Gut microbiota, Metagenome, Gastroenterology, Microbiology

## Abstract

Irritable bowel syndrome (IBS) is a common functional gastrointestinal disorder characterized by abdominal pain, altered bowel habits, and frequent comorbidity with anxiety and depression. The gut microbiota has been implicated in gut-brain axis (GBA) dysfunction, but consistent microbial signatures remain unclear. We performed whole metagenome shotgun sequencing of stool samples from 63 female patients with moderate to severe IBS and 34 female healthy controls and assessed microbial composition and functional pathways. Microbial richness and diversity were slightly reduced in IBS, though with high variability and no robust separation from controls. Differential abundance analyses revealed enrichment of *Streptococcus sp.* and the sulfate-reducing bacterium *Desulfovibrio piger* in IBS, alongside reductions in *Bifidobacterium* and *Methanobrevibacter*. Functional profiling identified 39 differentially abundant pathways: amino acid biosynthesis (e.g., L-isoleucine, L-threonine) was more prominent in IBS, while carbohydrate degradation pathways (e.g., galactose, stachyose) were enriched in healthy controls. These findings indicate modest but significant IBS-associated shifts in gut microbial composition and function that may contribute to IBS symptoms. However, high intra-group variability underscores the complexity of IBS and highlights the need for larger, multi-omics studies to define robust microbial markers. These results contribute to a growing body of evidence emphasizing the complexity of gut microbiota-host interactions and the need for high-resolution, systems-level approaches in microbiome-associated disorders.

**Supplementary Information:**

The online version contains supplementary material available at 10.1038/s41598-026-52163-w.

## Introduction

Irritable bowel syndrome (IBS) is a chronic gastrointestinal disorder of gut-brain interaction which affects approximately 4-10% of the population, with higher prevalence in females^1,2^. Symptoms include abdominal pain, bloating, and disturbed bowel function in the absence of structural or organic pathology^3–5^. In addition to gastrointestinal symptoms, IBS is often accompanied by fatigue^6^, and psychological conditions such as anxiety and depression^7,8^. Evidence shows that psychological stressors can affect bowel function^8–12^ or conversely, bowel symptoms can in turn, affect behavior and mood^13^. This results in a heterogeneous and complex disease model for which there are still no identified distinct biological mechanisms or markers^14–16^. It has been demonstrated that signals in the gut generated by the gut microbiota, alter nerve signals (neurotransmitters such as serotonin, glutamate, acetylcholine, γ-aminobutyric acid (GABA)) and immune and hormonal (endocrine factors and enzymes) responses between the gut and the brain^17^. These findings have attracted the attention of researchers towards understanding the role of the microbiota in conditions associated with gut-brain axis (GBA) dysfunction, such as IBS^18^.

Recent gut microbiome studies in IBS research have attempted to identify bacterial profiles or “signatures” in the microbiota at the phyla or genera level^19^. Still, the identification of specific species that clearly contribute to IBS symptomology have rarely been successful. The inconsistency in sequencing and analysis methods, limited sample size, type of specimen, gut-brain-imaging protocols, lack of details on the use of probiotics and antibiotics and diet habits, and high heterogeneity between healthy controls (HC) have made the identification of IBS-associated bacterial signatures and gut-brain interactions challenging. For example, the PCR-based 16S ribosomal RNA (rRNA) is the most common microbiome assessment technique but it is limited in depth and scope for analysis of fecal microbiota and has the potential to be biased as it requires the design of specific primers to target known phyla and genera and is thereby not optimal for the discovery of previously unknown gut bacteria at the species level^20–22^.

In a meta-analysis, Pittayanon et al.^23^ systematically assessed studies conducted from 2011 to 2018 and reported IBS-specific subtypes based on intestinal microbiota characterized by an increase in families *Enterobacteriaceae, Lactobacillaceae*, and *Bacteroidales* and a decrease in genus *Bifidobacterium*, *Fecalibacterium*, and *Clostridiales* compared to healthy individuals^23^. Though most of the studies in this meta-analysis used 16S rRNA analysis, other studies utilized methods such as denaturing gel gradient electrophoresis (DGGE), fluorescent in situ hybridization (FISH), terminal restriction fragment length polymorphism (TRFLP), quantitative polymerase chain reaction (qPCR) and microarray. Shortly following this meta-analysis, a Swedish random population study on the fecal and mucosa-associated microbiome of IBS patients based on 16S rRNA analysis reported no IBS-specific bacterial signatures^24^. Subsequent metagenome studies have aimed to identify unique bacterial signatures across IBS cohorts based mostly on 16S methods. To date, the overall findings have been inconsistent, with high variability between methods and study subjects’ fecal bacterial composition^25–32^.

Other approaches are clearly necessary to detangle conditions related to the GBA, such as a more recent study which jointly evaluated both the fecal microbiome *and* the metabolome. Jeffery et al. have reported significant differences in the fecal microbiome and metabolomes of individuals with or without IBS.^33^ Several other studies have shown that the gut microbiota, rich with *Firmicutes* and *Bacteroidetes* phyla, plays a vital role in carbohydrate, protein, and fatty acid metabolism. Furthermore, it has been shown that the impaired production of anti-inflammatory short-chain fatty acids (SCFAs) is associated with IBS^34,35^.

During the last decade, research has provided an increasing amount of evidence that supports the theory that the gut microbiome influences not only gastrointestinal functions but behavior and cognition as well.^8,36–44^ Most of this evidence comes from studies which demonstrate that the microbiota and its metabolites are associated not only with digestion and gut motility but also with stress, emotional behavior, and pain modulation^45–47^. As the role of the gut microbiota in IBS remains inconclusive, further exploratory investigations are required to examine potential relationships between specific bacteria and symptoms in IBS. Recent advances in ultrahigh-resolution metagenomic sequencing enable simultaneous interrogation of microbial taxonomic diversity and functional metabolic capacity, providing opportunities to move beyond single-taxon associations toward systems-level understanding of microbiota–host interactions. Such approaches are particularly relevant for heterogeneous disorders of gut–brain interaction such as IBS, where microbial contributions may arise from distributed community functions rather than discrete microbial signatures.

Using metagenome shotgun sequencing, we comprehensively investigated the gut microbiome compositional alterations in IBS patients (n=63) and healthy controls (HC) (n=34). We hypothesized that the fecal bacterial composition of IBS patients differs from HCs. Therefore, our primary aim was to identify specific bacterial taxa that differed between IBS and HCs that could potentially be associated with microbial functional pathways that contribute to alterations of gut function seen in IBS. To accomplish this, we further investigated the functional features of identified bacterial species and their associated metabolic pathways to predict the role of microbiota in IBS pathogenesis and host metabolism.

## Materials and methods

### Participants

**Fig. 1:**
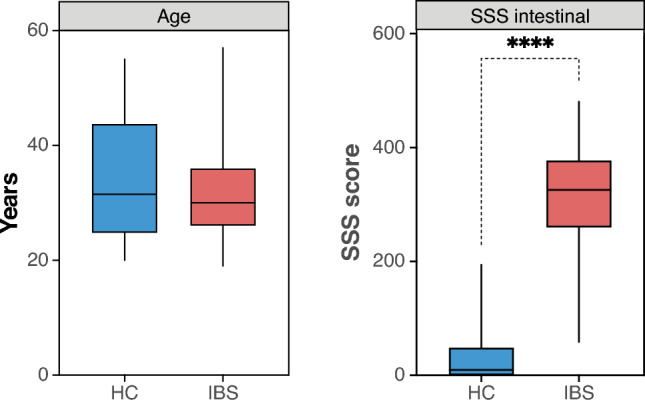
Demographic clinical data – Median of each variable stratified by a group for age and Severity of IBS-SSS intestinal symptoms(p<0.0001).

In total, 63 female patients with IBS, mean age of 32 years (range, 19-57 years), meeting Rome III criteria with mostly moderate to severe IBS, were analyzed from a larger patient cohort recruited from the Gastroenterology Department, University Hospital, Linköping, Sweden^48,49^. Healthy subjects (n=34) age-matched female controls (HC), a mean age of 33 years (range, 20-55 years), without a medical history of gastrointestinal symptoms or complaints, were recruited by advertisement. IBS patients were classified according to predominant bowel habits into IBS-mixed (IBS-M; n=36), IBS-constipation (IBS-C; n=12), and IBS-diarrhea (IBS-D; n=15). Exclusion criteria for both study groups were established via interviews with participants to determine if they suffered from any organic gastrointestinal disease, metabolic or neurological disorders, or severe psychiatric disease (e.g., schizophrenia, bipolar disorder.), as well as factors known to affect gut barrier function, including self-reported allergy, self-reported nicotine intake within the previous two months, and self-reported regular NSAID use. All participants were required to be fluent in Swedish. Due to the small number of male recruits as well as to avoid sex-related confounds, only female study participants were chosen for this study. All methods were performed in accordance with the relevant guidelines and regulations given by the Regional Ethical Review Board in Linköping which approved the study in the official registry (Dnrs. 2013/506-32; 2014/264-32), and all subjects gave their written informed consent. All patients’ information is summarized in Fig. 1 and Supplementary Table 1.

### Questionnaire data

#### Severity of IBS-SSS

IBS-SSS was used to evaluate the severity of IBS symptoms in both extra-and intra-intestinal categories, with five items measuring: frequency and intensity of abdominal pain, severity of bowel distension/bloating, satisfaction with bowel habits, and interference with daily life. Each item ranges between 0-100. A score of 75-175 corresponded to mild, 175-300 to moderate, and >300 points categorized as severe symptoms^50^.

### Microbiome analysis

#### Sample collection, library preparation and sequencing

**Fig. 2:**
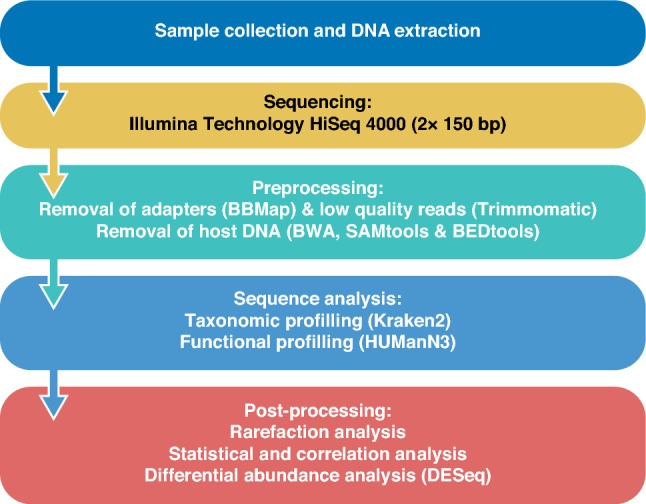
Schematic overview of the shotgun metagenomic analysis pipeline.

Stool samples were analyzed from all 97 participants (63 IBS and 34 HC). Frozen fecal samples were sent to GATC in 2019 (now Eurofins Genomics (Ebersberg, Germany)) for NexGen INVIEW Metagenome Explore-whole genome shotgun sequencing. Samples were thawed and underwent stool-optimized DNA extraction including cell wall lysis and elimination of compounds (e.g., digested food, bile acids, and bilirubin) before DNA extraction, purification, and normalization. DNA sequencing was performed using an Illumina Technology HiSeq 4000 (paired-end, 20 million reads, read mode 2 × 150 bp) Fig. 2.

The adapters were removed using BBMap (v38.18).^51^ The sequence reads were inspected for base quality from the 3´ and 5´ ends to remove low-quality reads. Trimmomatic^52^ filtered low-quality reads: LEADING:18 TRAILING:18 SLIDINGWINDOW:4:22 MINLEN:75. Bases with average Phred quality below 20 were considered low quality, and only mate pairs (forward and reverse read) were used for downstream analysis. Filtered reads were aligned to the GRCh37/hg19 genome using the BWA (v0.7.17)^53^ sequence aligner together with SAMtools (v1.19)^54^ and BEDtools (v2.30.0)^55^ to identify and remove human DNA sequences. After removing the host sequence reads, taxonomic profiling of the non-host sequences was performed using the Kraken2 version 2.0.8-beta^56^ and Minikraken2 v1 8GB database, including RefSeq bacteria, archaea and viral libraries. Kraken2 was selected as the preferred method after analyzing reads against kaiju and Metaphalan2 databases which assigned large proportion reads as “unclassified” at different taxonomic levels. Taxonomic classification performed with Kraken2 breaks the sequences into overlapping k-mers and is based on a 31-mer exact alignment. The k-mers are mapped to their lowest common ancestor of the genomes, containing that k-mer in a pre-computed reference database. Kraken2 output was then analysed using Bracken (Bayesian Re-estimation of Abundance with Kraken) (v2.5.0)^57^ to remove biases associated with reference genome selection and to estimate the true read abundance for each bacterial taxon.

#### Rarefaction

Rarefaction curves (R package vegan v2.5.6) were generated to determine whether all samples were sufficiently sequenced to represent their total bacterial identities. Reads were sub-sampled at every 2 M reads intervals to assess the saturation of samples.

#### Bioinformatics and statistical analysis

Alpha diversity estimates, including species richness (Observed), Shannon diversity, evenness and Chao1, were assessed using an estimate-richness function in the Vegan R package. Species richness gives the number of species observed in a sample. Wilcox-rank sum test evaluated differences in alpha diversity indices and species richness between the two study groups. Before subsequent compositional analyses, we applied a relative abundance cut-off of 0.001 to the metagenomic data. This reduced the taxon count to 1551 microbial species with relative abundance above 0.001.

Beta diversity or the compositional dissimilarity between the two groups was evaluated by Bray-Curtis dissimilarity. Samples were clustered using non-metric multidimensional scaling (NMDS), and Adonis (Permutation multivariate ANOVA—PERMANOVA) with 999 permutations was performed^58^ to evaluate differences in beta diversity using the Phyloseq and Vegan packages in R. sPLS-DA (sparse Partial Least Squares Discriminant Analysis), a supervised multivariate classification method, was applied to determine how a combination of microbial taxa best distinguishes predefined study groups (e.g., IBS vs healthy cohort). The k-fold (5-fold and 50-repeats) cross-validation method was applied to estimate the model’s classification errors using the perf() function in the mixOmicsR package. In addition, we generated a cross-validation error rate plot and a stability plot to validate the overfitting of the model. To assess the homogeneity of dispersion, we applied the betadisper function in the vegan R package, followed by a permutation test (999 permutations). Results were interpreted using PERMANOVA results to reflect the true differences in the microbial community composition.

#### Abundance measures and normalization

Abundance is measured by the count of taxa assigned reads from various taxonomic levels. Read counts of input samples observed at Phylum, Genus, and Species were normalized by using the centered log(2)-ratio (CLR) transformation^60^ implemented in the R package mixOmics^59^. Transforming compositional data using log ratios helps to reduce spurious correlations and allows sub-compositionally coherent comparisons between samples and study groups.^61,62^ The sparse PLS discriminant analysis (sPLS-DA) from the mixOmics package^59,63^ in R was utilized to detect the signature of discriminative taxa between the HCs and IBS patients.

#### Correlations

Using the lmer function in the lme4 package in R, we evaluated confounding factors affecting microbiota diversity and bacterial species abundance that were significantly altered in IBS patients compared with HCs. Associations between these factors were evaluated using Spearman’s rank correlation coefficient. To control for multiple testing, p-values were adjusted using the Benjamini–Hochberg procedure to estimate the false discovery rate (FDR). Spearman’s correlation (rho) with p < 0.1 was considered statistically significant.

#### Differential abundance

The differential abundance of microbial taxa between IBS patients and HCs was estimated using the DESeq2 method. This method estimates the taxon-wise dispersion by negative binomial likelihood for each feature. Hypothesis testing was performed using the Wald test, and the results were analyzed using Benjamin-Hochberg’s FDR-corrected p-values at 0.05.

#### Profiling of functional pathways

Functional metagenome annotation, including pathway and gene-family abundances, was conducted by HMP Unified Metabolic Analysis Network 3. This tool aligns the filtered reads to their pan-genomes of species in the sample detected by MetaPhlAn2 for each metagenome.^64^ By default, HUMAnN3 reconstructs the profile of metabolic pathways based on the gene families annotated to the MetaCyc database^65^ and gene families were annotated using a comprehensive protein database UniRef90^66^. Post-analysis was performed to combine the pathway abundance files from each sample using the “humann_join_tables” command, and combined data were normalized to copies-per-million (CPM) units using “humann_renorm_table”. The metabolic potential of gut bacteria was summarized across metabolic pathways, and the “humann2_associate” function was utilized to compare the pathway abundances between the two study groups.

## Results

### Study subjects

Participants were categorized into two main groups: IBS individuals (n=63) and healthy cohort (n=34), and all downstream analyses were performed only on those two groups. No additional sub-categories based on the severity of IBS or based on bowel habits were considered for sequence analyses due to the limited number of samples in each sub-group.

#### Sequencing results

In total, 97 samples entered the shotgun metagenomic analysis pipeline. The average proportion of filtered reads was 13.1 million per sample with a standard deviation of 4.1 million. The average proportion of filtered reads assigned as “Unclassified’ was 62%, where only around 38% of total reads were mapped to known bacterial species. The rarefaction curve plots the number of species against the number of samples, showed that expected bacterial species richness increased with the number of reads and reached a plateau for most samples at around 4 million reads (Supplementary Figure S1). In total, 30 phyla, 63 classes, 260 families and 675 genera consisting of both bacteria and archaea were identified in all samples.

#### Gut microbiota diversity and richness measures

**Fig. 3:**
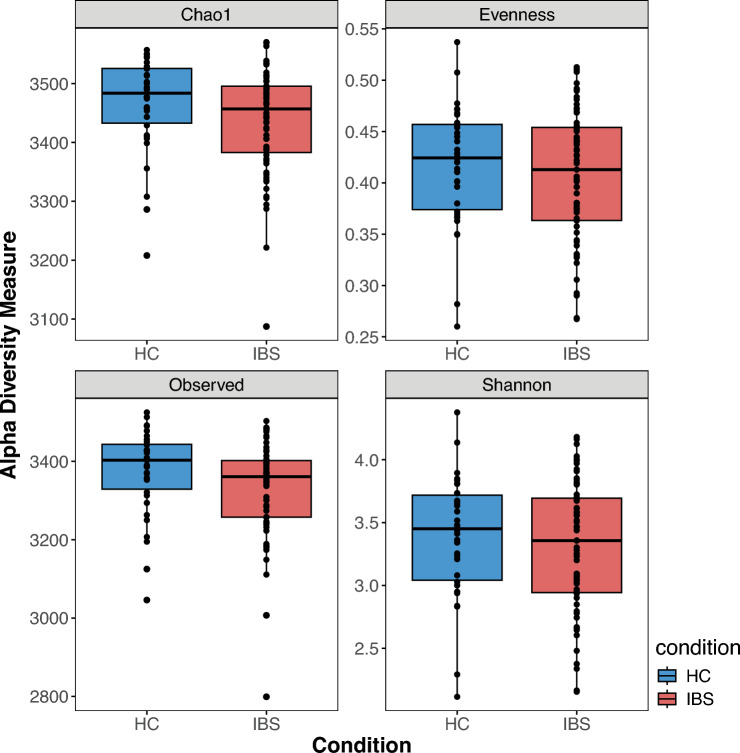
Distribution of alpha diversity estimates. Chao1 richness, Pielou’s evenness, and Observed and Shannon indices stratified by groups HCs and IBS.

A total of 3686 taxa were observed in HCs and IBS patients after filtering out the bacterial species that were not present in at least 50% of samples to capture the true representation of taxa and reduce any false positives in the dataset. A slightly higher number of taxa (Chao1 richness) was observed in the HC group (median 3403) than in the IBS (median 3361). Except for the Observed number of taxa (Wilcoxon rank sum test p-value < 0.05), none of the other alpha diversity estimates were statistically significantly different between the two groups (Wilcoxon rank sum test p-value > 0.05) (Fig. 3).

#### Beta diversity: compositional dissimilarity between IBS patients and the healthy cohort

**Fig. 4:**
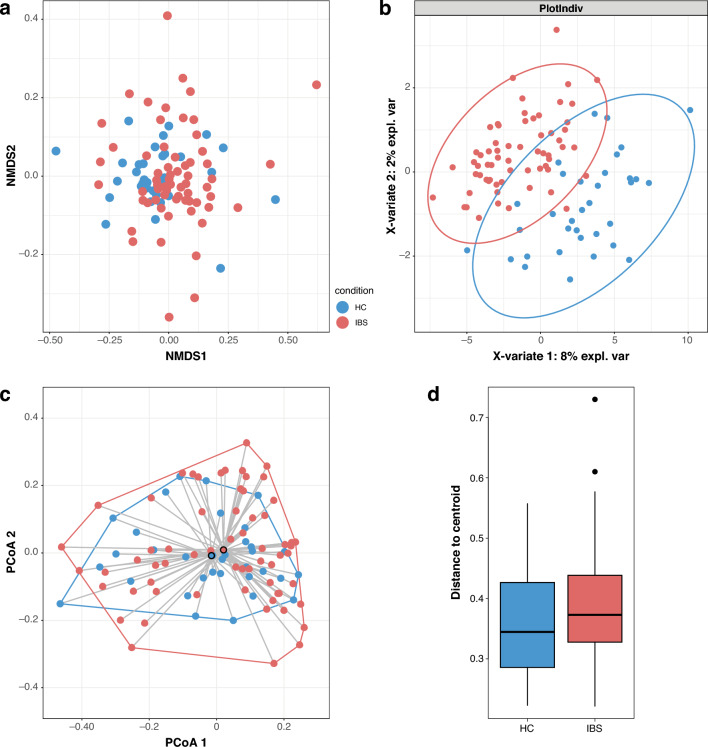
**a**, Non-metric multidimensional scaling plot displaying the compositional differences between the two study groups. **b**, Supervised Partial Least Squares Discriminant Analysis (sPLS-DA) analysis, showing the discrimination between the sample groups. **c,** Principal coordinates analysis (PCoA) with convex hulls and distances to group centroids, visualizing the spread of samples within each group. **d,** Betadispersion analysis (distance to centroid) comparing within-group variability.

To assess differences in microbial community composition between IBS patients and healthy controls (HCs), NMDS analysis of Bray–Curtis distances was performed. No significant difference in overall community composition was observed between groups (PERMANOVA, p > 0.05), and substantial overlap was evident in the ordination (Fig. 4a). To evaluate within-group variability, we performed a betadispersion analysis, which indicated a trend toward greater dispersion in the IBS group compared to HCs (mean distance to centroid: IBS = 0.39; HC = 0.35), although this difference did not reach statistical significance (permutation test, p = 0.061; Fig. 4d). This pattern is consistent with the broader spread of IBS samples in the ordination space but does not provide statistical support for increased heterogeneity. Similarly, sPLS-DA with 0.95 confidence ellipses showed substantial overlap between groups and did not reveal robust discrimination (Fig. 4b). Model performance assessment indicated a relatively low classification error rate and a limited number of stable taxa, suggesting that the model was not strongly overfitting (Supplementary Figure S2 a,b).

**Fig. 5:**
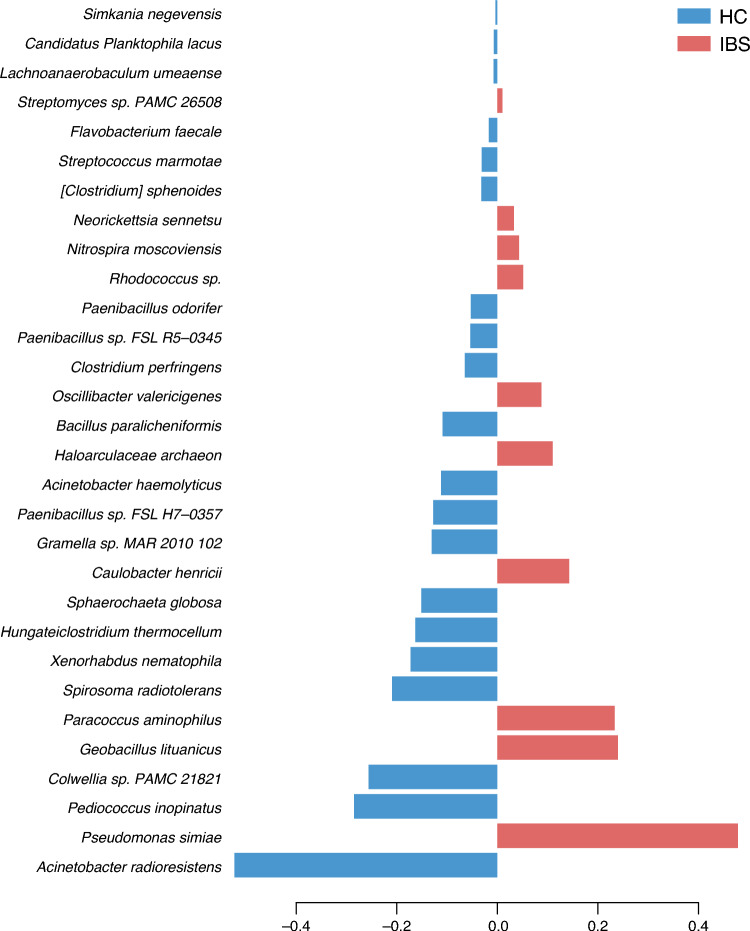
Contributions to component 1 from the Supervised Partial Least Squares Discriminant Analysis (sPLS-DA) analysis, showing the discrimination between the sample groups.

As per the bacterial species loading plot, 30 bacterial signatures from phyla and families *Proteobacteria* (*Moraxallaceae, Pseudomonadaceae, Rhodobacteriaceae*)*, Firmicutes* (*Lactobacilliaceae, Bacilliaceae*) and *Bacteroidetes* (*Cytophagaceae*) were identified as the contributors of component 1 (Fig. 5).

In healthy individuals, *Pseudomonas simiae*, *Geobacillus litunicus*, *Paracoccus aminophillus*, *Spirosoma radiotolerans*, *Caulobacter henricii*, *Haloarculaceae archaeon*, *Oscillibacter valericigenes*, *Rhodococcus_sp*., *Nitrospira moscoviensis*, *Neorickettsia sennetsu*, and *Streptomyces_sp._PAMC_26508* were the most abundant taxa. The key bacterial signatures that discriminated between the two groups were *Acinetobacter radioresistens*, *Pseudomonas simiae, Pediococcus inopinatus, and Colwellia sp. PAMC 21821, Geobacillus litunicus, Paracoccus aminophillus, and Spirosoma radiotolerans* (i.e., the 6 largest contributions at the bottom of Fig. 5). The results are similar to the findings reported in previous studies.^67^

#### Gut microbial composition: relative abundance measures

**Fig. 6:**
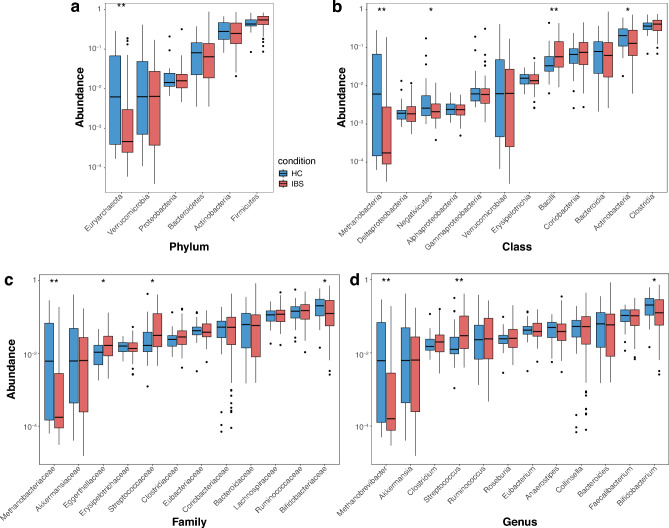
Between-group comparison of relative abundances of the most abundant **a**, phyla; **b**, classes; **c**, families; and **d**, genera. *p <0.05, **p <0.01.

Across both groups, the most abundant bacterial phyla were *Firmicutes*, *Actinobacteria*, *Bacteroidetes, Verrucomicrobia*, *Euryarchaeota*, and *Proteobacteria,* which together made up approximately 80% of total abundance (Fig. 6a). The remaining were among other phyla with relative abundance below 0.01. The phylum-level abundance values highly varied among samples, yet the relative abundance of the phylum *Euryarchaeota* (Kingdom Archaea) was significantly lower in patients with IBS relative to HCs. The remaining dominant phyla showed a relatively consistent distribution between the two study groups.

At the class level, the highest median of relative abundance was for *Clostridia*, followed by *Actinobacteria*, *Bacteroidia*, and *Bacilli*. The relative abundance of remaining classes were below 1% (Fig 6b). The abundance of *Bacilli* was significantly higher in IBS patients while *Methanobacteria*, *Negativicutes*, and *Actinobacteria* were lower in IBS patients relative to HCs.

At the family level, *Bifidobacteriaceae*, *Ruminococcaceae*, *Lachnospiraceae* and *Bacteroidaceae* were the highly abundant bacterial families in both IBS patients and healthy individuals (Fig. 6c). IBS patients showed a significantly higher abundance of *Streptococcaceae and Eggerthellaceae,* and significantly lower abundance of *Methanobacteriaceae* and *Bifidobacteriaceae* at the family level*.*

At the genus level, *Bifidobacterium* (phylum *Actinobacteria*), *Fecalbacterium* (phylum *Firmicutes*), *Bacteroides* (phylum *Bacteroidetes*), *Collinsella* (phylum *Actinobacteria*), *Streptococcus* (phylum *Firmicutes*), *Ruminococcus* (phylum *Firmicutes*), *Anaerostipes* (phylum *Firmicutes*), *Eubacterium* (phylum *Firmicutes*), and *Akkermansia* (phylum *Verrucomicrobiota*), were the most prevalent and abundant across all samples although a majority of genera were observed with a higher abundance in patients with IBS compared to HCs (Fig. 6d). According to the relative abundance values, the Archaea genus *Methanobrevibacter* and *Bifidobacterium* presented a significantly lower abundance in IBS, while *Streptococcus* was significantly higher when compared to HCs. Particularly the genus *Methanobrevibacter* is the most prevalent archaea taxa in a healthy gut microbiome.

#### Differential abundance analysis - Dseq2

The difference in taxa abundance between IBS and HCs has been considered a potential indicator of altered gut microbiome composition in IBS pathogenesis. Hence, we conducted differential abundance analyses to identify statistically significantly different taxa between the two study groups.

#### Differential genera abundance

We found 23 differentially abundant bacterial genera in the two groups. There were 14 genera in IBS patients and 9 in healthy individuals. Out of these 23 genera, only two genera, *Streptococcus* and *Lactobacillus* from phylum *Firmicutes* were relatively high in abundance (above 0.1%) and were significantly higher in abundance in patients with IBS (p < 0.05). The remaining were rare taxa (relative abundance below 0.1%) from the genus *Dialister* (phylum *Firmicutes*) and *Oxolobacter* (phylum *Proteobacteria*) which were highly abundant in HCs (Supplementary Table S3).

#### Differential species abundance

**Fig. 7:**
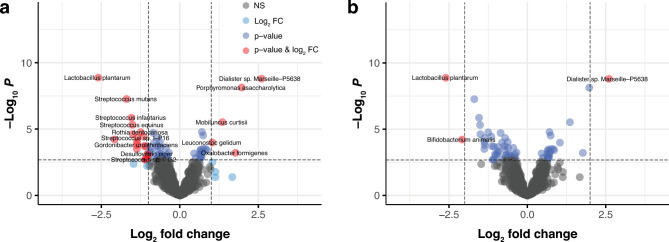
Differentially abundant species. **a**, Log_2_ FC > 1 and p-value < 0.05. 24 bacterial species were sampled. **b**, Log_2_ FC > 2 and *p* < 0.05. 3 bacterial species were sampled.

Although species-level abundances were reported in very low values, we extended the differential abundance analysis to the species level. Here we found 68 bacterial species that were significantly different (p-value < 0.05) between IBS and HCs (Supplementary Table S3). Species were considered strongly different when their abundance in HCs was at least twofold higher than in IBS, corresponding to a log₂ fold change greater than 1(Log2 Fold Change > 1). Here, 24 of those 68 bacterial species passed the abundance threshold shown by a red dot on the volcano plot (Fig. 7) and in Supplementary Table S2.

Species that were dominant and differentially abundant in HCs were *Dialister_sp._Marseille-P5638, Porphyromonas_asaccharolytica, Oxalobacter_formigenes*., *Mobiluncus_curtisii,* and *Leuconostoc_gelidum.* Abundance profiles of these species showed that *Lactobacillus plantarum*, *Bifidobacterium animalis* and *Streptococcus mutans* were significantly reduced in abundance in healthy females compared to IBS patients. Moreover, Actinobacterial species *Rothia dentocariosa* and *Gordonibacter urolithinfaciens* and Proteobacteria species *Desulfovibrio piger* were also found differentially abundant in the members of the IBS cohort. Interestingly, most bacterial species mentioned above are generally common inhabitants in the human gut or mucosa or have been clinically important as pathogenic agents. Also, we noted that some probiotic bacterial strains such as *Bifidobacterium animalis*, *Lactobacillus plantarum*^68^ and *Lactobacillus gasseri* which play an important role in host metabolism were relatively high in abundance in IBS patients compared to HCs.

#### Validation between sPLS-DA and Deseq2

For validation between sPLS-DA and Deseq2 methods, we compared the 30 bacterial signatures which potentially discriminated between IBS an HC groups in the sPLS-DA analysis (Fig. 5), to the 68 species identified in the Deseq2 differential species method (Supplementary Table S3). Here we found that 17 of these species were overlapping between the different analytical approaches we used to identify differential bacterial signatures between IBS and HCs (Table 1). This group of bacterial species may indicate a potential group of discriminative bacterial species that differentiate the gut microbiota composition in IBS patients from healthy individuals found using two complimentary methods. Interestingly, none of the 24 bacterial species that passed both the abundance p-value and abundance threshold in the Deseq2 method (Fig. 7 & Supplementary Table S2) agreed with the 30 found in the sPLS-DA analysis, indicating that the 17 found in agreement between the two methods were likely not highly abundant species in the total sample.

**Table 1:**
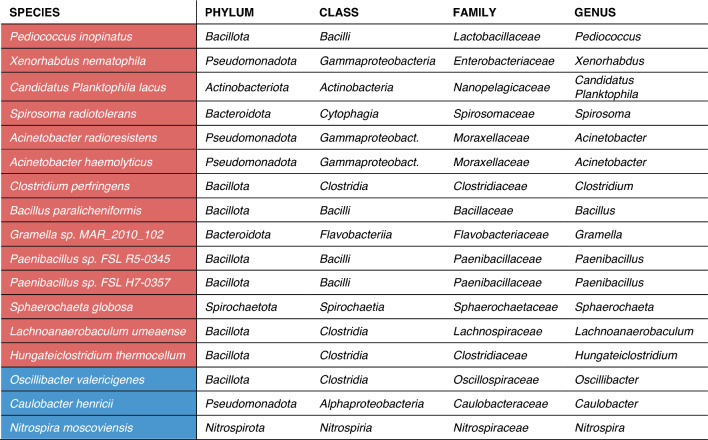
Seventeen species that differentiate IBS (red) from HC (blue) in both the sPLS-DA and Deseq2 methods.

#### Gut microbial functional pathways in IBS and HCs

The potential functionality of gut microbiota in patients with IBS and HCs was annotated using the MetaCyC database using HuMAnN3. Results showed that approximately 60% of filtered reads were classified as unmapped and unintegrated against the MetaCyC database. Within the classified reads, the most abundant pathways in all participants combined were lactose and galactose degradation (LACTOSECAT-PWY), adenosine and guanosine ribonucleotides de novo biosynthesis (PWY-7219; PWY-7221), starch degradation V (PWY-6737), L-valine biosynthesis (VALSYN-PWY), L-isoleucine biosynthesis I (ILEUSYN-PWY) and glycolysis IV (PWY-1042). The dominant pathways identified in both groups involved amino acid biosynthesis, nucleoside and nucleotide biosynthesis, carbohydrate biosynthesis and cofactor, carrier, and vitamin biosynthesis. Typically, these pathways are governed by the function of healthy gut bacterial communities. Our metagenome compositional analysis indicated that several species of *Lactobacillus*, *Bifidobacterium* and *Streptococcus* were found to among the top 12 most abundant genera in both IBS patients and HCs. In general, these lactic-acid bacteria are considered probiotic strains and may exhibit a favorable impact on gut regularity and digestion, inflammation, and gut immune system. The HuMAnN3 results summarized that these bacterial groups are potentially related to amino acid, carbohydrate, and energy metabolism. For example, valine biosynthesis, glycolysis, tryptophan biosynthesis, lactose and galactose degradation and starch degradation are a few of the characterized functional pathways.

Based on these results, and to maintain analyses on the more abundant features of the general microbiome, we then extended our analysis to the most differentially abundant pathways, mapped the functional features of those pathways to their species-specific biomarkers within each sample, and then stratified the findings to the respective groups. Between the two study groups, we noticed 39 differentially abundant pathways (Wilcoxon rank sum test p-value < 0.1) (Supplementary Table S4). Enriched levels in the super pathway of L-threonine biosynthesis (THRESYN-PWY: Amino acid biosynthesis) and the super pathway of L-isoleucine biosynthesis I (PWY-3001: Amino acid biosynthesis) were found significantly higher in patients in IBS group, while thiamine phosphate formation from pyrithiamine and oxythiamine (PWY-7357: Amino acid biosynthesis), stachyose degradation (PWY-6527: Carbohydrate degradation) and D-galactose degradation I (PWY-6317; PWY66-422: Carbohydrate degradation) were enriched in healthy individuals (Supplementary Figures S3-S8). Similar to previous reports, we also noticed a high abundance of amine-related metabolic pathways in IBS patients.

Within the members of the IBS group, differentially abundant super pathway L-isoleucine biosynthesis I (PWY-3001: Amino acid biosynthesis) were found to be mainly served by Streptococci (Phylum Firmicutes) species, including *Streptococcus salivarius, Streptococcus thermophilus, Bifidobacteria lognum, and Bifidobacteria bifidum* (Supplementary Figure S5). These four bacterial species, although highly prevalent and abundant in IBS patients, did not significantly differ between IBS and HC groups.

The enriched D-galactose degradation I metabolic features identified in HCs were mainly hosted by *Firmicutes* species *Anaerostipes hadrus, Coprococcus eutactus, Ruminococcus torques, Roseburia intestinalis* as shown in (Supplementary Figure S4), these bacteria are relatively abundant across all healthy individuals compared to patients with IBS. The significantly abundant genus *Dialister* and *Leuconostoc* species were found to be associated with many functional pathways but mainly with amino acid biosynthesis such as L-lysine biosynthesis, and folate transformations III (E. coli) in HCs. Our metagenome functional analysis revealed 39 differential metabolic pathways between IBS patients and HCs and results indicated that microbial metabolic pathways are not led by a single member of the gut microbiota but by various members of the commensal microbiota. Our results further suggest that both microbiome and metabolic features can be used as potential biological features by which to differentiate IBS patients from HCs and find potential remedies to IBS pathogenesis.

## Discussion

This study compared the composition of the gut microbiome of 97 female participants with IBS (n=63) to HCs (n=34). Literature suggests that IBS pathogenesis is linked with either reduced or altered bacterial diversity and richness^33,69,70^. In our metagenomic study, although we observed a slight reduction in bacterial richness and diversity values in the IBS group relative to HCs, we were unable to robustly distinguish the two groups to a significant degree. Our diversity measures revealed high intra-group variability, which agrees with many reports in the literature^71,72^ and is often the explanation given for a lack of robust differentiation between groups.

The betadispersion analysis indicated a trend toward greater inter-individual variability within the IBS group compared to healthy controls, consistent with the broader spread of IBS samples observed in the NMDS ordination. Although this difference did not reach statistical significance, sPLS-DA likewise failed to demonstrate clear separation between groups, suggesting that between-subject variability exceeds group-level differences in this dataset. In this context, IBS-associated differences are best described as modest shifts in the relative abundance of multiple taxa and metabolic pathways rather than a single dominant microbial signature. While the cross-sectional nature of the data precludes inference about coordinated or dynamic community behavior, the concurrent compositional and functional differences observed across multiple features are consistent with a distributed pattern of microbiome alteration rather than a single defining taxonomic marker.

According to many theories, the gut microbiota plays a key role in IBS, but existing studies have shown conflicting results due to inconsistencies in cohort sizes, intra-group variability, sampling protocols, and methods of analysis^23,33,73–78^. Although we observed no clear separation between IBS patients and HCs in the measures of gut microbiota richness or diversity indices, by analyzing differential abundances, we identified several bacterial taxa which potentially act as IBS-associated bacterial signatures. Unlike previous 16S studies, our metagenome results did not find a significant phylum-level difference in *Bacteroidetes* or *Firmicutes* abundance between the IBS and HCs, but rather a significant difference in the abundance of the phylum *Euryarchaeota,* which was significantly lower in IBS patients compared to HCs. IBS patients also showed a significantly higher abundance in the genera *Streptococcus* and lower abundance of *Bifidobacterium* and *Methanobrevibacter* when compared to healthy participants^79–81^. At the species level we found 68 differentially abundant bacterial species, 17 of which were found to similarly differentiate IBS from HCs using two separate methods of analysis, both sPLS-DA and Deseq2.

Of the bacterial species found to be differentially abundant in IBS, some are commensal and commonly found in the human microbiome such as *Lachnoanaerobaculum umeaense*^82^, but others have been implicated in overgrowth and disease particularly in immunocompromised individuals such as *Acinetobacter haemolyticus* and *Clostridium perfringens*^83,84^. Of the group of 24 differentially abundant species in Supplementary Table S2, some have been implicated in oral diseases, including *Streptococcus mutans and australis, Rothia dentocariosa*, and *Actinomyces oris*^85–87^*.* Those more abundant in IBS which have been implicated in bowel disease include *Desulfovibrio piger,* and *Streptococcus infantarius and equinus* (*S. bovis complex*)^88–91^. ^88,89^Notably, some bacterial strains such as *Bifidobacterium animalis*, *Lactobacillus plantarum* and *Lactobacillus gasseri* are thought to play an important role in host metabolism and are widely found in fermented foods and probiotics^92^, and were also relatively high in abundance in IBS patients compared to HCs.

Both sPLS-DA and DESeq2 provide complementary perspectives on microbial community differences. DESeq2 identifies taxa that are individually differentially abundant between groups using a univariate framework, whereas sPLS-DA captures combinations of taxa whose joint variation contributes to group discrimination through multivariate co-abundance patterns. As such, the two approaches are inherently comparable in their aim to detect group-associated features, but differ in the structure of the signals they are designed to identify. The limited overlap between taxa identified by these methods likely reflects these methodological differences: DESeq2 is sensitive to strong, consistent changes in individual taxa, while sPLS-DA can detect weaker but coordinated variation across multiple taxa that may not reach significance in isolation. Additionally, the high inter-individual variability and modest effect sizes observed in this dataset may further reduce concordance between univariate and multivariate approaches. Taxa identified uniquely by sPLS-DA should therefore not be dismissed as false positives, but rather interpreted as candidate features reflecting community-level structure, albeit with caution given the potential for model overfitting and the need for independent validation.

We additionally examined microbiome functional metabolic pathways and found 39 differentially abundant pathways between the two groups (Supplementary Table S4). IBS microbiota functional synthesis pathways were found to be abundant in probiotic strains and lactic-acid producers such as *Lactobacillus plantarum, Streptococcus mutans, Streptococcus infantarius, Streptococcus equinus, Streptococcus parasanguinis*, Actinobacterial species *Rothia dentocariosa* and *Gordonibacter urolithinfaciens* and Proteobacteria species *Desulfovibrio piger*. Many of these species have been found to be abundant and commensal in the healthy human gut microbiome, as they are functionally linked with amino acid synthesis, carbohydrate degradation, and SCFAs such as acetate, butyrate, and propionate production that provide energy to intestinal epithelial cells and enhance the gut barrier function, yet some commensal microbes can still act as opportunistic pathogens in dysbiosis. Interestingly, a significantly high abundance of a major sulfate-reducing bacteria *Desulfovibrio piger* indicates possible changes in metabolic output in IBS patients^90^. This further suggests that excessive sulfide production could influence the intestinal structure, regularity, and digestion and contribute to IBS pathogenesis^91^.

The lactic-acid-producing bacteria Streptococcus thermophilus and potentially Streptococcus salivarius have been reported to produce GABA via glutamate decarboxylase in a strain-dependent manner. While microbially derived GABA has been proposed to contribute to gut–brain signaling, its physiological relevance in humans remains unclear. Although increased abundance of Streptococcus has been observed in IBS cohorts, the functional implications for enteric nervous system (ENS) activity are not yet established^93^. In the present study, the higher relative abundance of Streptococcus in IBS samples may be consistent with altered microbial metabolic potential but does not provide direct evidence of ENS modulation.

The species Collinsella aerofaciens is involved in carbohydrate metabolism, including lactose utilization and fermentation, and has been linked to bile acid metabolism and host–microbe interactions^94^. Although Collinsella abundance varied across samples and was higher in IBS patients in this cohort, the factors underlying this difference remain unclear. While these taxa may contribute to the functional pathways identified, such as amino acid biosynthesis, our results do not support attribution of these pathways to individual species, but rather suggest that they arise from the combined activity of multiple members of the microbial community. Although we identified 39 metabolic pathways that differed between IBS and HCs, their heterogeneous and low-abundance profiles limited our ability to pinpoint specific bacterial drivers of IBS pathogenesis and symptoms. Understanding such distributed functional alterations may inform future microbiota-targeted strategies, including dietary modulation or probiotic interventions, even when taxonomic biomarkers alone show limited discriminatory power.

As metagenomic analysis moves toward clinical application, the field still faces challenges in resolving microbes at fine taxonomic scales. The gut microbiota is an exceptionally diverse and metabolically active ecosystem, and advances in high-throughput sequencing are only beginning to reveal its intricate relationship with human health. By combining complementary analytical approaches with metagenomic profiling, as demonstrated in this study, we can begin to identify key metabolic features of the microbiome that distinguish health from disease. Looking ahead, the integration of multi-omics data linking microbiome, metabolome, and patient symptomology and physiology holds great promise for unraveling the microbial and metabolic mechanisms underlying complex disorders such as IBS.

## Limitations

Although we observed some significant differences in gut microbiota abundance and metabolic pathway abundances between IBS patients and HCs, we were only able to classify 38% of the whole-genome readouts and 62% of reads remained “unclassified”. The classified proportion is relatively low, and our statistics indicate the partiality of reference databases on previously sequenced genomes and the lack of sensitivity in classification algorithms for detecting novel microbes. A major limitation of this study relates to the metagenomic taxonomic classification pipeline used at the time of analysis (2019). The analyses were performed using Kraken2 (v2.0.8 beta) in combination with the MiniKraken database, reflecting the state of available tools at that time. While the core classification algorithm of Kraken2 has remained stable, both software versions and reference databases have since undergone substantial development. In addition, related tools used for taxonomic and functional profiling, such as HUMAnN and MetaPhlAn and their associated reference databases, have also been significantly updated, and these advances are not reflected in the current results. Limited current knowledge pertaining to the functional features of IBS-related bacteria also restricts our understanding of their metabolic role in IBS progression and overall gut health. In addition, greater sample size and documentation of concurrent use of probiotics or diet would have been essential in understanding between-group differences between IBS patients and healthy participants. Lastly, although the focus on high-severity females with IBS reduces group and sex-related confounds, it limits generalizability to the IBS in low-severity and male subjects. Our study highlights the need for future studies that focus more on the clinical investigation of gut microbiota and metabolites to understand the potential routes of IBS pathophysiology.

## Supplementary Information

Supplementary Information.

## Data Availability

The raw sequencing data have been submitted to the European Nucleotide Archive (ENA), accessible via https://www.ebi.ac.uk/ena/browser/view/PRJEB34103 under project accession ID: PRJEB34103.
