# Supplementary material for: High-resolution metagenomic characterization of gut microbiota composition and functional pathways in irritable bowel syndrome
Source: Sci Rep. 2026 May 20;16:15742. doi: 10.1038/s41598-026-52163-w (PMC13190689; doi:10.1038/s41598-026-52163-w)
Supplement: Supplementary file 1 — Supplementary Information. [file 41598_2026_52163_MOESM1_ESM.pdf]

SUPPLEMENTARY INFORMATION

Characterization of gut microbiota in patients with IBS and healthy controls using whole metagenome shotgun sequencing

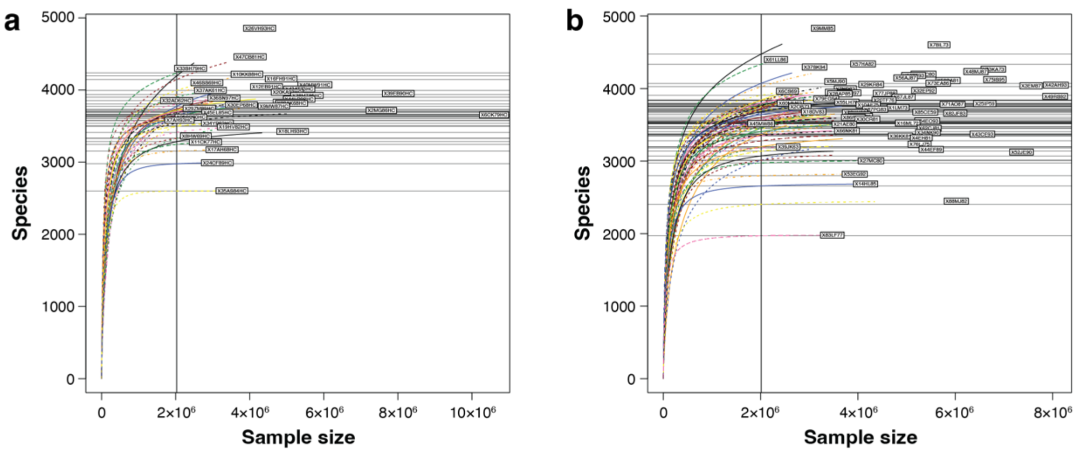

Supplementary Figure S1. Rarefaction curves of species vs sample size **a.** Healthy controls, **b.** IBS.

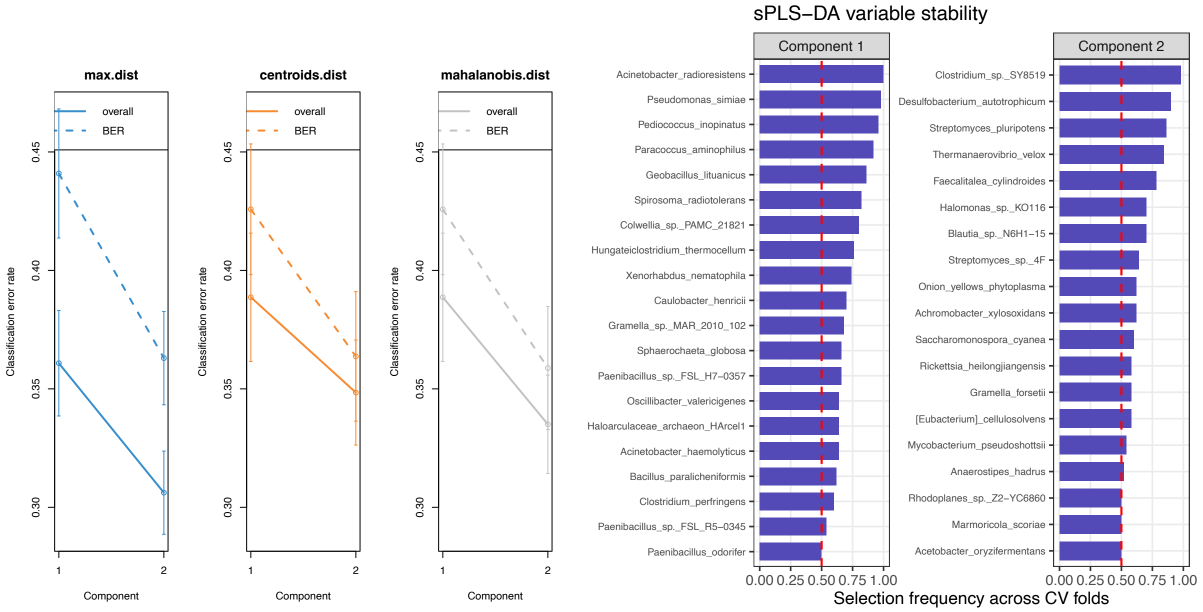

Supplementary Figure S2. **a.** Cross-validated error rate plot: This shows the classification error rate and the balanced error rate (BER) as a function of the number of components. The values are between 0.35 and 0.4, which is relatively low and may provide evidence against overfitting.
b. Variable stability plot: This plot shows the selection frequency of each taxon across cross-validation folds. Out of all taxa included in the analysis, 20 showed selection frequencies ≥ 0.5 and were considered stable for component 1, while the remaining taxa (250 or more) fell below this threshold and were excluded from biological interpretation. We assume that if the model were severely overfit, many taxa would appear stable at high frequency.

Genera	baseMean	Log_2 Fold Change	lfcSE	Stat	p-value	p_{adj}
Streptococcus	236158.0982	-1.435291791	0.371951019	-3.858819351	0.000113936	0.011129875
Coriobacterium	531.0364278	-1.172461725	0.282242783	-4.154089298	3.27E-05	0.006255079
Gordonibacter	10441.24544	-0.991029286	0.257032773	-3.855653399	0.000115421	0.011129875
Lactobacillus	16149.11202	-0.96512227	0.279385818	-3.454442596	0.000551432	0.024814422
Actinomyces	3500.549822	-0.700006941	0.209163162	-3.346702801	0.000817788	0.029667818
Cronobacter	39.34045291	-0.696374933	0.19898641	-3.49961051	0.000465938	0.023917998
Aliivibrio	108.3155878	-0.691680896	0.187732209	-3.68440185	0.00022924	0.015888985
Calditerrivibrio	41.11480977	-0.666773786	0.200764845	-3.321168038	0.000896415	0.029667818
Kyrpidia	60.19322687	-0.569975124	0.177214707	-3.216296959	0.001298564	0.0398423
Xenorhabdus	116.2124107	-0.541557105	0.143663982	-3.76960944	0.000163503	0.013795583
Photobacterium	173.2298605	-0.457673081	0.118666084	-3.856814551	0.000114874	0.011129875
Lachnoanaerobaculum	2340.139955	-0.433861364	0.124230241	-3.492397348	0.000478706	0.023917998
Halobacillus	224.035904	-0.413976758	0.129545681	-3.195604488	0.001395382	0.040951442
Sphaerochaeta	364.1957828	-0.323072618	0.087847563	-3.677650305	0.000235392	0.015888985
Desulfomicrobium	148.6920745	0.432555681	0.130303954	3.319589827	0.000901498	0.029667818
Gemmata	34.14823297	0.594633503	0.178589995	3.329601427	0.000869704	0.029667818
Thermaerobacter	146.7948213	0.597006619	0.177471699	3.363953933	0.000768344	0.029667818
Cystobacter	53.17921532	0.624646172	0.179348333	3.482865784	0.000496077	0.023917998
Thiocystis	45.38803267	0.727144734	0.176275829	4.125039365	3.71E-05	0.006255079
Actinomadura	56.85895332	0.807059338	0.224528408	3.594464268	0.00032506	0.019946848
Mobiluncus	58.22246051	1.300524482	0.28485256	4.565605729	4.98E-06	0.001680933
Oxalobacter	279.9720045	1.712781439	0.516987538	3.313003339	0.000922999	0.029667818
Dialister	5509.210134	2.372734891	0.399677445	5.936624448	2.91E-09	1.96E-06

Supplementary table S1. 23 Differentially abundant genera that passed Log2 Fold change >1. Those are the red-dots on the volcano plot (figure 5)

Species	Phylum	Base Mean	log2 Fold Chng	lfcSE	stat	p-value	p_{adj}	HC	IBS
Dialister sp. Marseille-P5638	Bacillota/Firmicutes	5084.93	2.6050	0.4316	6.0359	0.0000	0.0000	High	
Porphyromonas asaccharolytica	Bacteroidota	115.19	1.9725	0.3411	5.7836	0.0000	0.0000	High	
Oxalobacter formigenes	Proteobacteria	281.90	1.7692	0.5167	3.4243	0.0006	0.0239	High	
Mobiluncus curtisii	Actinomycetota	59.88	1.3557	0.2902	4.6714	0.0000	0.0008	High	
Leuconostoc gelidum	Bacillota/Firmicutes	193.01	1.0375	0.2673	3.8806	0.0001	0.0081	High	
Lactobacillus plantarum	Bacillota/Firmicutes	450.29	-2.6001	0.4287	-6.0650	0.0000	0.0000		High
Bifidobacterium animalis	Actinomycetota	3042.65	-2.0818	0.5191	-4.0104	0.0001	0.0059		High
Streptococcus mutans	Bacillota/Firmicutes	280.08	-1.6939	0.3116	-5.4360	0.0000	0.0000		High
Streptococcus infantarius	Bacillota/Firmicutes	189.98	-1.5413	0.3192	-4.8278	0.0000	0.0004		High
Streptococcus equinus	Bacillota/Firmicutes	881.32	-1.5101	0.3301	-4.5750	0.0000	0.0011		High
Streptococcus salivarius	Bacillota/Firmicutes	15620.84	-1.4972	0.3545	-4.2236	0.0000	0.0034		High
Lactobacillus delbrueckii	Bacillota/Firmicutes	952.13	-1.3717	0.3804	-3.6058	0.0003	0.0164		High
Streptococcus sp. FDAARGOS 192	Bacillota/Firmicutes	832.68	-1.3648	0.3499	-3.9003	0.0001	0.0081		High
Gordonibacter urolithinfaciens	Actinomycetota	3926.63	-1.3639	0.3607	-3.7819	0.0002	0.0115		High
Streptococcus sp. I-P16	Bacillota/Firmicutes	326.01	-1.3587	0.3336	-4.0729	0.0000	0.0048		High
Rothia dentocariosa	Actinomycetota	305.32	-1.2475	0.2903	-4.2980	0.0000	0.0030		High
Coriobacterium glomerans	Actinomycetota	534.45	-1.1489	0.2784	-4.1270	0.0000	0.0044		High
Lactobacillus sakei	Bacillota/Firmicutes	144.29	-1.1355	0.3041	-3.7342	0.0002	0.0130		High
Streptococcus parasanguinis	Bacillota/Firmicutes	7784.57	-1.1067	0.3590	-3.0828	0.0021	0.0475		High
Streptococcus sp. I-G2	Bacillota/Firmicutes	272.14	-1.0939	0.3528	-3.1005	0.0019	0.0462		High
Actinomyces oris	Actinomycetota	1409.04	-1.0728	0.2892	-3.7092	0.0002	0.0134		High
Streptococcus australis	Bacillota/Firmicutes	680.69	-1.0384	0.3354	-3.0956	0.0020	0.0462		High
Desulfovibrio piger	Thermodesulfobacteriota	485.42	-1.0317	0.3064	-3.3675	0.0008	0.0267		High
Lactobacillus gasseri	Bacillota/Firmicutes	176.99	-1.0107	0.3051	-3.3123	0.0009	0.0285		High

Supplementary table S2. 24 Differentially abundant species that passed Log2 Fold change >1. These are the red markers on the volcano plot (Figure 6).

Species	Phylum	S	baseMean	log2 FoldΔ	lfcSE	stat	p-value	p-adj
Lactobacillus plantarum	Bacillota/Firmicutes		450.2892	-2.6001	0.4287	-6.0650	0.0000	0.0000
Bifidobacterium animalis	Actinomycetota		3042.6504	-2.0818	0.5191	-4.0104	0.0001	0.0059
Streptococcus mutans	Bacillota/Firmicutes		280.0844	-1.6939	0.3116	-5.4360	0.0000	0.0000
Streptococcus infantarius	Bacillota/Firmicutes		189.9752	-1.5413	0.3192	-4.8278	0.0000	0.0004
Streptococcus equinus	Bacillota/Firmicutes		881.3165	-1.5101	0.3301	-4.5750	0.0000	0.0011
Streptococcus salivarius	Bacillota/Firmicutes		15620.8386	-1.4972	0.3545	-4.2236	0.0000	0.0034
Lactobacillus delbrueckii	Bacillota/Firmicutes		952.1305	-1.3717	0.3804	-3.6058	0.0003	0.0164
Streptococcus sp. FDAARGOS 192	Bacillota/Firmicutes		832.6846	-1.3648	0.3499	-3.9003	0.0001	0.0081
Gordonibacter urolithinfaciens	Actinomycetota		3926.6289	-1.3639	0.3607	-3.7819	0.0002	0.0115
Streptococcus sp. I-P16	Bacillota/Firmicutes		326.0053	-1.3587	0.3336	-4.0729	0.0000	0.0048
Rothia dentocariosa	Actinomycetota		305.3231	-1.2475	0.2903	-4.2980	0.0000	0.0030
Coriobacterium glomerans	Actinomycetota		534.4485	-1.1489	0.2784	-4.1270	0.0000	0.0044
Lactobacillus sakei	Bacillota/Firmicutes		144.2935	-1.1355	0.3041	-3.7342	0.0002	0.0130
Streptococcus parasanguinis	Bacillota/Firmicutes		7784.5723	-1.1067	0.3590	-3.0828	0.0021	0.0475
Streptococcus sp. I-G2	Bacillota/Firmicutes		272.1436	-1.0939	0.3528	-3.1005	0.0019	0.0462
Actinomyces oris	Actinomycetota		1409.0419	-1.0728	0.2892	-3.7092	0.0002	0.0134
Streptococcus australis	Bacillota/Firmicutes		680.6927	-1.0384	0.3354	-3.0956	0.0020	0.0462
Desulfovibrio piger	Thermodesulfobacteriota		485.4230	-1.0317	0.3064	-3.3675	0.0008	0.0267
Lactobacillus gasseri	Bacillota/Firmicutes		176.9900	-1.0107	0.3051	-3.3123	0.0009	0.0285
Streptococcus constellatus	Bacillota/Firmicutes		104.4235	-0.9667	0.2884	-3.3520	0.0008	0.0270
Vibrio alginolyticus	Proteobacteria		47.0771	-0.9377	0.2540	-3.6915	0.0002	0.0138
Streptococcus parauberis	Bacillota/Firmicutes		219.1640	-0.9365	0.2670	-3.5069	0.0005	0.0201
Gordonibacter pamelaeae	Actinomycetota		5540.7548	-0.9339	0.2714	-3.4405	0.0006	0.0239
Pediococcus inopinatus	Bacillota/Firmicutes	Y	57.0314	-0.8529	0.1995	-4.2742	0.0000	0.0030
Clostridium autoethanogenum	Bacillota/Firmicutes		63.4552	-0.8090	0.2085	-3.8810	0.0001	0.0081
Streptococcus lutetiensis	Bacillota/Firmicutes		408.1802	-0.7963	0.2565	-3.1043	0.0019	0.0462
Xenorhabdus nematophila	Proteobacteria	Y	46.0531	-0.7269	0.1950	-3.7284	0.0002	0.0130
Candidatus Planktophila lacus	Actinobacteria	Y	58.6786	-0.7160	0.2288	-3.1295	0.0018	0.0445
Spirosoma radiotolerans	Bacteroidota	Y	33.5916	-0.7143	0.1994	-3.5817	0.0003	0.0168
Acinetobacter radioresistens	Proteobacteria	Y	76.6437	-0.7079	0.1737	-4.0746	0.0000	0.0048
Acinetobacter haemolyticus	Proteobacteria	Y	34.2094	-0.7028	0.2124	-3.3088	0.0009	0.0285
Cronobacter muytjensii	Proteobacteria		39.2142	-0.6646	0.1994	-3.3326	0.0009	0.0278
Calditerrivibrio nitroreducens	Deferribacterota		40.5023	-0.6330	0.1968	-3.2158	0.0013	0.0360
Clostridium perfringens	Bacillota/Firmicutes	Y	1695.9667	-0.6285	0.1608	-3.9078	0.0001	0.0081
Halobacillus mangrovi	Bacillota/Firmicutes		81.2015	-0.6190	0.1689	-3.6657	0.0002	0.0146
Bacillus paralicheniformis	Firmicutes	Y	87.0415	-0.5924	0.1758	-3.3700	0.0008	0.0267
Kyrpidia tusciae	Bacillota/Firmicutes		59.8448	-0.5522	0.1763	-3.1315	0.0017	0.0445
Actinomyces radicidentis	Actinomycetota		73.6767	-0.5520	0.1703	-3.2422	0.0012	0.0347
Gramella sp. MAR 2010 102	Bacteroidota	Y	63.2434	-0.5505	0.1664	-3.3083	0.0009	0.0285
Mucilaginibacter mallensis	Bacteroidota		64.0698	-0.5390	0.1753	-3.0740	0.0021	0.0482
Paenibacillus sp. FSL R5-0345	Bacillota/Firmicutes	Y	102.6796	-0.4997	0.1572	-3.1791	0.0015	0.0388
Paenibacillus sp. FSL H7-0357	Bacillota/Firmicutes	Y	191.0752	-0.4484	0.1226	-3.6573	0.0003	0.0146
Sphaerochaeta globosa	Spirochaetes	Y	163.0607	-0.4087	0.1270	-3.2184	0.0013	0.0360
Lachnoanaerobaculum umeaense	Bacillota/Firmicutes	Y	2311.1086	-0.4040	0.1210	-3.3399	0.0008	0.0277
Clostridium aceticum	Bacillota/Firmicutes		269.5236	-0.3664	0.1182	-3.0999	0.0019	0.0462
Hungateiclostridium thermocellum	Bacillota/Firmicutes	Y	348.3044	-0.3495	0.1019	-3.4287	0.0006	0.0239
Spirochaeta thermophila	Spirochaetes		111.9388	0.4545	0.1338	3.3958	0.0007	0.0259
Desulfomicrobium orale	Proteobacteria		73.5630	0.5070	0.1593	3.1819	0.0015	0.0388
Thermaerobacter marianensis	Bacillota/Firmicutes		149.7180	0.6304	0.1877	3.3592	0.0008	0.0269
Aminomonas paucivorans	Synergistota		48.6254	0.6443	0.2080	3.0983	0.0019	0.0462
Gemmata obscuriglobus	Planctomycetota		34.5688	0.6467	0.1836	3.5218	0.0004	0.0196
Corynebacterium variabile	Actinomycetota		37.7533	0.6570	0.2016	3.2587	0.0011	0.0334
Cystobacter fuscus	Myxococcota		54.0680	0.6668	0.1864	3.5771	0.0003	0.0168
Streptomyces sp. SCSIO 03032	Actinomycetota		34.6770	0.6671	0.2094	3.1865	0.0014	0.0388
Ruminococcus champanellensis	Bacillota/Firmicutes		5191.6275	0.6908	0.2010	3.4368	0.0006	0.0239
Luteimonas sp. 83-4	Proteobacteria		31.3454	0.6924	0.2148	3.2230	0.0013	0.0360
Oscillibacter valericigenes	Bacillota/Firmicutes	Y	2586.8069	0.7041	0.1635	4.3069	0.0000	0.0030
Deinococcus gobiensis	Deinococcota		67.5754	0.7058	0.2093	3.3718	0.0007	0.0267
Acetobacter aceti	Proteobacteria		41.2308	0.7180	0.2067	3.4738	0.0005	0.0221
Thiocystis violascens	Proteobacteria		45.5184	0.7426	0.1781	4.1702	0.0000	0.0039
Caulobacter henricii	Proteobacteria	Y	37.0294	0.7456	0.2071	3.6008	0.0003	0.0164
Actinomadura amylolytica	Actinomycetota		57.3260	0.8204	0.2278	3.6015	0.0003	0.0164
Nitrospira moscoviensis	Nitrospirota	Y	35.8372	0.8562	0.2411	3.5517	0.0004	0.0180
Leuconostoc gelidum	Bacillota/Firmicutes		193.0054	1.0375	0.2673	3.8806	0.0001	0.0081
Mobiluncus curtisii	Actinomycetota		59.8784	1.3557	0.2902	4.6714	0.0000	0.0008
Oxalobacter formigenes	Proteobacteria		281.8993	1.7692	0.5167	3.4243	0.0006	0.0239
Porphyromonas asaccharolytica	Bacteroidota		115.1920	1.9725	0.3411	5.7836	0.0000	0.0000
Dialister sp. Marseille-P5638	Bacillota/Firmicutes		5084.9302	2.6050	0.4316	6.0359	0.0000	0.0000

Supplementary table S3. Deseq2 resulting 68 bacterial species that were significantly different (p-value < 0.05) between IBS and healthy controls. **S** column indicates which agreed with sPLS-DA analysis.

Feature	IBS abundance (mean)	Healthy abundance (mean)	P-value	Q-value
PWY-7357: thiamine phosphate formation from pyrithiamine and oxythiamine (yeast)	1.176e+04	7088	0.01784	0.05697
PWY-6317: D-galactose degradation I (Leloir pathway)	1.089e+04	6698	0.07029	0.1881
PWY66-422	1.089e+04	6698	0.07029	0.1881
PWY-6527: stachyose degradation	1.101e+04	6585	0.05815	0.1645
PWY-3001: superpathway of L-isoleucine biosynthesis I	9586	4614	0.001562	0.01966
THRESYN-PWY: superpathway of L-threonine biosynthesis	9693	4261	0.0007559	0.01966
GLUTORN-PWY: L-ornithine biosynthesis I	1.559e+04	1.7e+04	0.01278	0.04687
PWY-6121: 5-aminoimidazole ribonucleotide biosynthesis I	1.591e+04	1.73e+04	0.003876	0.01966
PWY-6122: 5-aminoimidazole ribonucleotide biosynthesis II	1.591e+04	1.73e+04	0.003876	0.01966
PWY-6277: superpathway of 5-aminoimidazole ribonucleotide biosynthesis	1.591e+04	1.73e+04	0.003876	0.01966
PWY-6386: UDP-N-acetylmuramoyl-pentapeptide biosynthesis II (lysine-containing)	1.591e+04	1.73e+04	0.003971	0.01966
PWY-6737: starch degradation V	1.589e+04	1.73e+04	0.003515	0.01966
DTDPRHAMSYN-PWY: dTDP-β-L-rhamnose biosynthesis	1.591e+04	1.731e+04	0.003876	0.01966
ILEUSYN-PWY: L-isoleucine biosynthesis I (from threonine)	1.591e+04	1.731e+04	0.003876	0.01966
PEPTIDOGLYCANSYN-PWY: peptidoglycan biosynthesis I (meso-diaminopimelate containing)	1.591e+04	1.731e+04	0.003876	0.01966
PWY-5686: UMP biosynthesis I	1.591e+04	1.731e+04	0.003876	0.01966
PWY-6387: UDP-N-acetylmuramoyl-pentapeptide biosynthesis I (meso-diaminopimelate containing)	1.591e+04	1.731e+04	0.003876	0.01966
PWY-7219: adenosine ribonucleotides de novo biosynthesis	1.591e+04	1.731e+04	0.003876	0.01966
PWY-7221: guanosine ribonucleotides de novo biosynthesis	1.591e+04	1.731e+04	0.003876	0.01966
VALSYN-PWY: L-valine biosynthesis	1.591e+04	1.731e+04	0.003876	0.01966
PWY-7400: L-arginine biosynthesis IV (archaebacteria)	1.559e+04	1.72e+04	0.00303	0.01966
PWY-6151: S-adenosyl-L-methionine salvage I	1.591e+04	1.727e+04	0.003971	0.01966
PWY-1042: glycolysis IV	1.589e+04	1.726e+04	0.005171	0.02226
PWY-6385: peptidoglycan biosynthesis III (mycobacteria)	1.588e+04	1.726e+04	0.003783	0.01966
PWY-6163: chorismate biosynthesis from 3-dehydroquinate	1.591e+04	1.725e+04	0.004481	0.02113
NONMEVIPP-PWY: methylerythritol phosphate pathway I	1.591e+04	1.709e+04	0.01172	0.04464
PWY-5097: L-lysine biosynthesis VI	1.571e+04	1.686e+04	0.005818	0.024
PWY-6700: queuosine biosynthesis I (de novo)	1.534e+04	1.682e+04	0.002673	0.01966
PWY-6609: adenine and adenosine salvage III	1.556e+04	1.668e+04	0.004931	0.02219
PWY-7111: pyruvate fermentation to isobutanol (engineered)	1.591e+04	1.661e+04	0.01582	0.05399
TRNA-CHARGING-PWY: tRNA charging	1.414e+04	1.643e+04	0.001438	0.01966
PWY-5103: L-isoleucine biosynthesis III	1.559e+04	1.634e+04	0.01907	0.05899
PWY-3841: folate transformations II (plants)	1.562e+04	1.605e+04	0.03257	0.09771
PWY-4242	1.545e+04	1.599e+04	0.01667	0.055
NONOXIPENT-PWY: pentose phosphate pathway (non-oxidative branch) I	1.474e+04	1.533e+04	0.0386	0.1124
TRPSYN-PWY: L-tryptophan biosynthesis	1.387e+04	1.51e+04	0.01422	0.05029
PWY-5667: CDP-diacylglycerol biosynthesis I	1.499e+04	1.512e+04	0.07615	0.1933
PWY0-1319: CDP-diacylglycerol biosynthesis II	1.499e+04	1.512e+04	0.07615	0.1933
PWY-5695: inosine 5'-phosphate degradation	1.129e+04	1.333e+04	0.01106	0.04378

Supplementary table S4. 39 differentially abundant pathways (Wilcoxon rank sum test p-value < 0.1)

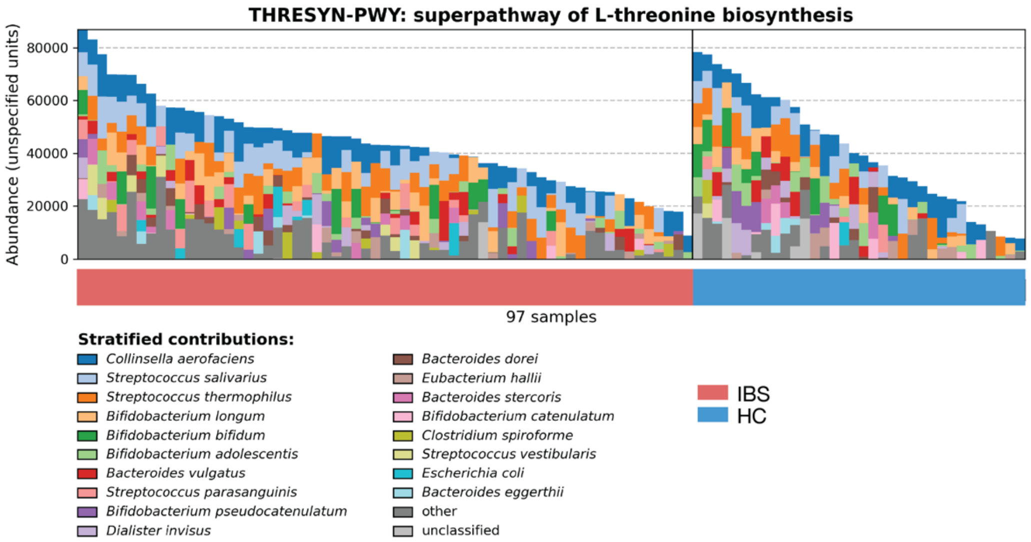

Supplementary Figure S3. Abundance levels for THRESYN-PWY.

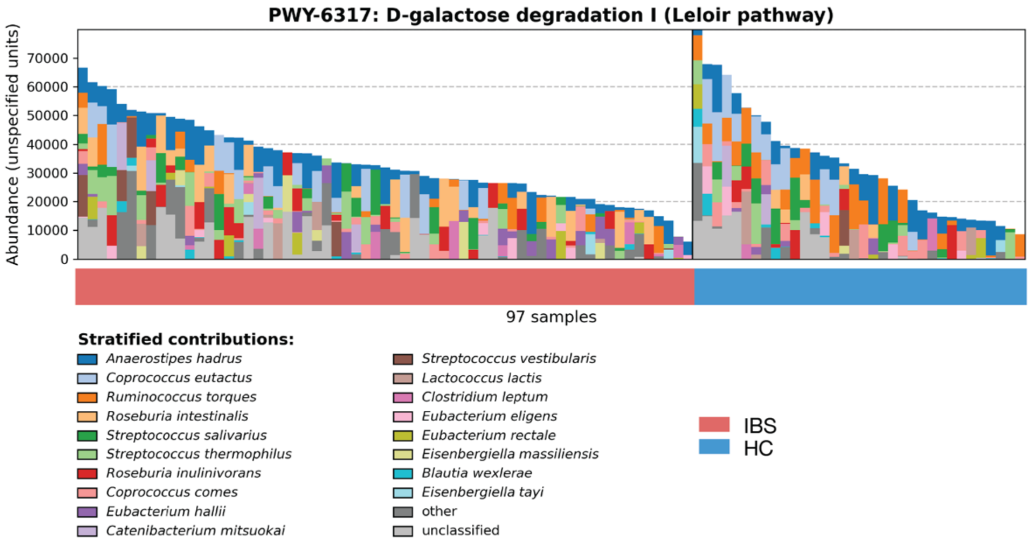

Supplementary Figure S4. Abundance levels for PWY-6317.

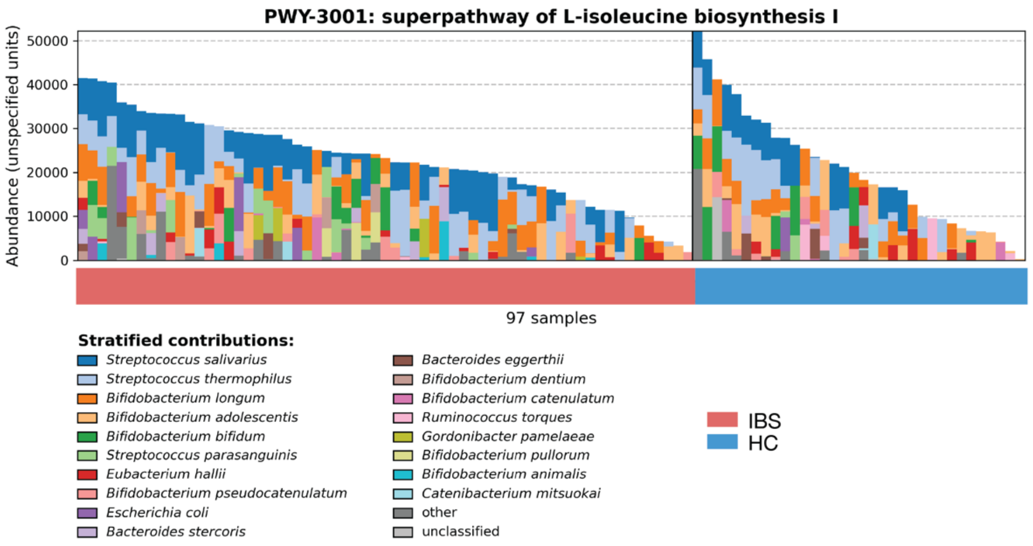

Supplementary Figure S5. Abundance levels for PWY-3001.

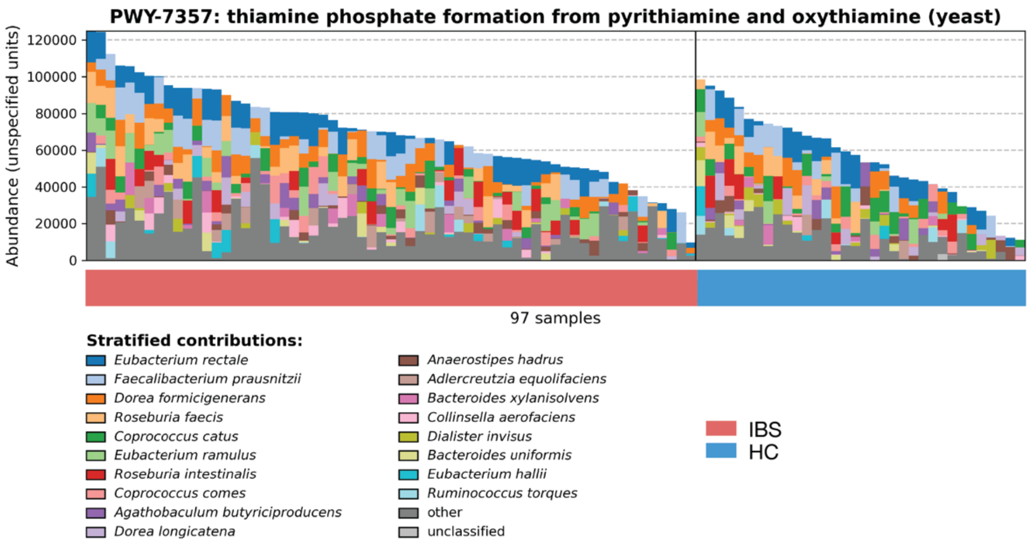

Supplementary Figure S6. Abundance levels for PWY-7357.

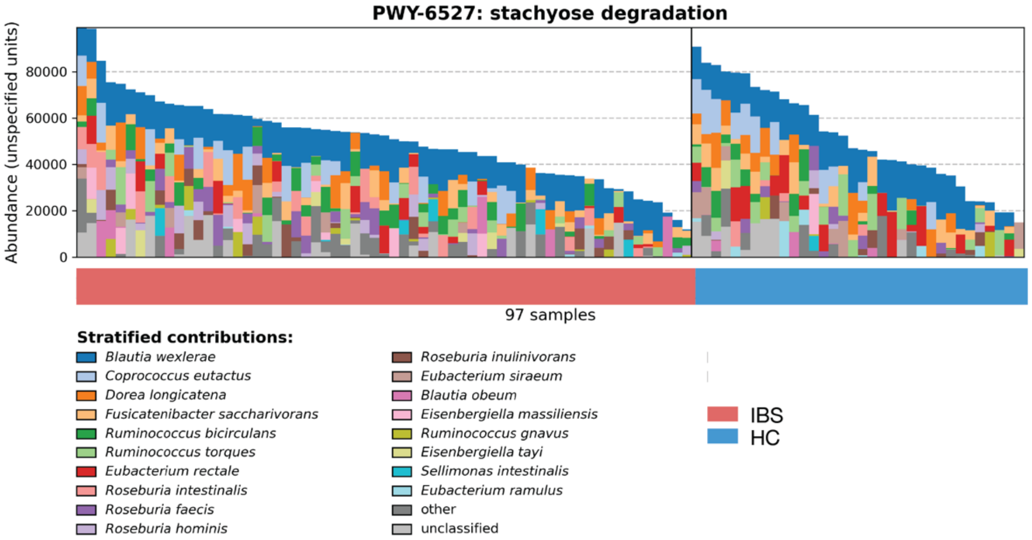

Supplementary Figure S7. Abundance levels for PWY-6527.

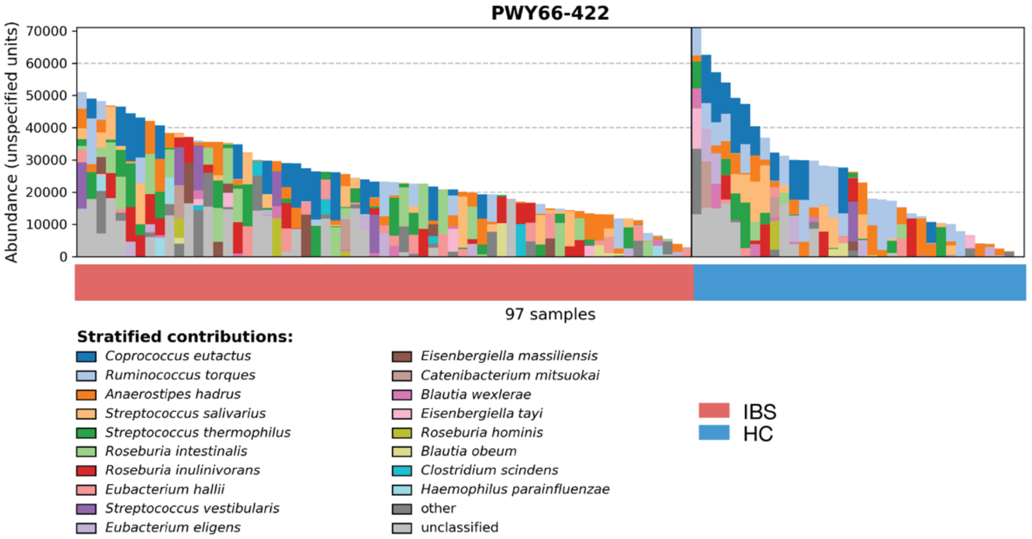

Supplementary Figure S8. Abundance levels for PWY66-422.
